# Supplementary material for: GDBM: A database of global drainage basin morphology
Source: PLoS One. 2025 Apr 7;20(4):e0320771. doi: 10.1371/journal.pone.0320771 (PMC11975112; doi:10.1371/journal.pone.0320771)
Supplement: Attachment — Submitted filename: response.pdf [file pone.0320771.s001.pdf]

Queen Mary
University of London

Stuart W. D. Grieve
School of Geography
Queen Mary University of London
Mile End Rd, London
E1 4NS
Phone: +44 (0)20 7882 2780
Email: s.grieve@qmul.ac.uk

February 4, 2025

Dear Editor,

Thank you for considering our revised manuscript 'GDBM: A database of global drainage basin morphology' for publication in PLOS ONE.

We would like to take the opportunity to thank you and the two reviewers for your continued engagement in our work. We believe that in revising our manuscript in line with the comments and suggestions provided, we are now submitting a manuscript with a renewed clarity in its terminology, its place in the wider literature, and its scientific rigour. The new manuscript has a revised introduction and abstract which better place our work within the wider research context, additional technical detail on how we generate the dataset and clearer description of the uses and limitations of the basin measurements within the dataset.

This document outlines the changes to the manuscript we have made, with each reviewer's comments in **bold type** and our responses in standard type. In the document below, wherever we refer to a Figure or Equation in our responses, we are referring to the numbers in the revised manuscript. Following our responses, a copy of the manuscript with our changes highlighted is enclosed.

Sincerely,

Stuart W. D. Grieve

Editorial requests

1. Please ensure that your manuscript meets PLOS ONE's style requirements, including those for file naming.
We have prepared the manuscript using the PLOS ONE LaTeX template and believe that we have followed all formatting guidelines. If there are specific outstanding issues which need to be addressed, please let us know.

2. We note that Figure 1 in your submission contain [map/satellite] images which may be copyrighted. All PLOS content is published under the Creative Commons Attribution License (CC BY 4.0), which means that the manuscript, images, and Supporting Information files will be freely available online, and any third party is permitted to access,

download, copy, distribute, and use these materials in any way, even commercially, with proper attribution. For these reasons, we cannot publish previously copyrighted maps or satellite images created using proprietary data, such as Google software (Google Maps, Street View, and Earth).
We can confirm that all of the data presented in Figure 1 is CC BY 4.0. The data presented in Figure 1a is drawn from an open access dataset - [*Peel et al.*, 2007] - which is appropriately cited within the manuscript. This dataset was released through the Journal Hydrology and Earth System Sciences, which releases its material under a CC BY 4.0 licence, as shown here: HESS Copyright Guidelines.

The data in Figure 1b is similarly drawn from an open access dataset, described within these papers, which are cited within the manuscript: [*Zomer et al.*, 2008; *Trabucco et al.*, 2009]. The specific data files are provided under a CC BY 4.0 licence via Figshare.

Reviewer 1

I have thoroughly examined the manuscript titled "GDBM: A database of global drainage basin morphology " The study aims to present a new near-Global dataset of Drainage Basin Morphology, GDBM, which provides morphometric measurements of 254,966 basins based on Shuttle Radar Topography Mission (SRTM) topographic data with Köppen-Geiger climate classifications. The authors have done commendable work; however, before getting accepted, the manuscript needs major revision to strengthen the impact of the research. Kindly see the following comments.
We thank the reviewer for taking the time to review our manuscript and a pleased that they recognise the potential of our work to have a positive impact on our field.

1. Although the abstract claims that the dataset was generated by combining Köppen-Geiger climatic classifications with SRTM topography data, it doesn't explain exactly how this was done.

Thanks for flagging up this area of confusion. We have revised the relevant portion of the abstract to more clearly outline the process by which this dataset is created, with the aim of making it more explicit what steps we take to generate the data presented in this manuscript:

> *This dataset is created by extracting channels from the 30-meter resolution Shuttle Radar Topography Mission (SRTM) topographic data which fall within Köppen-Geiger climate zones, to allow the influence of climate on river and basin morphology to be quantified.*

Additionally, channels were classified with "high confidence," however it's not obvious how this was determined.

The concept of 'high confidence' is discussed in detail in the manuscript (Lines 129 to 136 in the revised manuscript), and refers to the idea that because we are using a very conservative drainage area threshold, we are minimising the number of false positive channels within the dataset.

We have also revised the relevant section of the abstract to provide more clarity on this topic:

> *These data have been generated with minimal assumptions, focusing on identifying and*

classifying channels with high confidence, through the use of a conservative drainage area threshold.

2. In the abstract, novelty is not given enough attention.

In line with the editorial guidelines of PLOS ONE, we have focused our abstract and manuscript as a whole on presenting our dataset with scientific rigour, rather than focusing on advertising the novelty of our work as it's defining criteria. However, we believe that in addressing the review comments we have received as a whole, the abstract and manuscript does now do a better job of clearly stating the novelty and potential impact of our work.

3. The abstract needs a strong conclusion regarding the potential impact and applications of the dataset.

Thank you for this suggestion. We have expanded the end of the abstract to better outline potential uses to interested readers:

> *GDBM provides opportunities for rapid spatial analysis of channel morphology at a near-global scale and has the potential to yield continuing insight into landscape evolution across diverse climate regimes. This dataset also has potential applications across a range of Earth and environmental science domains, through the integration of additional data on, for example, forest canopy height, landcover, or soil properties to explore the spatial variability of channel and basin properties with climate.*

4. Using a drainage threshold of 22.5 km^2 may still introduce some bias, especially in areas with naturally low drainage network densities (such as deserts). Defend (justify) it.

We do not dispute that the selection of a particular threshold is always going to be a compromise, but are confident that our conservative approach maximises the likelihood of true positives and minimises false positives within our channel extraction scheme. Conceptually, as we decrease the drainage threshold the channel network we extract becomes denser, extending further upslope. As identified in this comment, this would be a particular concern in landscapes with lower drainage density, such as arid landscapes. We present sensitivity analyses in this manuscript which demonstrate that modifying the threshold does not meaningfully alter the population of metrics that we are computing, suggesting that although there is no globally correct threshold area for channel extraction, the threshold we are using is appropriate for our use case and is not unduly influencing the dataset we are presenting here.

5. The dataset is intimately linked to estimates of worldwide aridity and Köppen-Geiger climatic zones, providing possibilities to examine river and basin morphology in relation to climate. However, it could be difficult to distinguish morphological changes caused by the environment from those resulting from other causes like geology or human activity.

We agree that at a local scale it is challenging to disentangle direct causes for the morphology of individual rivers due to the competition between natural and human processes. However, the power of our dataset is in its size, which allows large scale patterns in channel morphology to be identified across a wide population of channels. For example, we have demonstrated a climate-concavity signal *Chen et al.* [2019] and climate-basin morphology signal *Singer et al.* [2024] using the GDBM dataset. In performing these analyses, we manually excluded data across several categories (last glacial maximum

extent, tectonic activity, sand dunes) and found that all of the climate-morphometric relationships identified in the full dataset hold even when excluding these data, giving us high confidence that our results are not unduly impacted by these second order effects (See Table S4 in *Singer et al.* [2024] for details, reproduced in the appendix of this response for convenience).

6. The use of the dataset is restricted by the exclusion of the polar climatic zones (ET and EF). Explain the exclusion.

This dataset was created to understand relationships between climate and fluvial channel morphology, and so we have excluded polar regions where channels are highly likely to have been modified by ice and related non-fluvial processes. The SRTM dataset which underpins GDBM also does not include data in polar regions. This is why we describe our dataset as 'near global' and outline these constraints in full in the manuscript:

> *Due to the nature of the space shuttle's orbit, topographic data was only collected between 60°N and 56°S Farr et al. [2007]. This limits the creation of a truly global dataset, however, as the purpose of these data are to understand the relationships between climate and fluvial channel morphometry, excluding polar data where channel forms will be carved by ice is beneficial.*

7. The accuracy of the dataset in capturing dryland hydrological dynamics may be negatively affected by differences in the resolution of climatic zone data and Aridity Index data.

We agree that this is a potential limitation of our dataset, and articulate this point within the manuscript in Section 3.4. Our solution to this potential limitation is to provide metadata about the aridity value sampling within GDBM, thereby allowing users to filter the dataset according to their needs. For example, it is therefore possible to filter based on the number of aridity measurements along a channel, or their standard deviation, to exclude channels where the aridity signal is inconsistent along the channel length.

8. While channel extraction from Shuttle Radar Topography Mission (SRTM) data at 30-meter resolution is a well-established approach in geomorphology; however, it has drawbacks compared to modern LiDAR data. Give a reason for using the SRTM data.

We chose to use SRTM data to underpin this study for a number of reasons: 1) The data is freely available and has complete spatial coverage of the parts of the world where we wished to perform our analysis; 2) The dataset has been used extensively in geomorphology research, and has been thoroughly validated and evaluated by the community, creating a solid foundation for us to build on; and 3) SRTM data at 30m resolution provides a good compromise between computational efficiency and spatial resolution.

We agree that LiDAR data has many advantages over SRTM data, however, this is a near-global scale dataset and global coverage LiDAR datasets are not available. This would leave us creating a dataset where we could only identify channels in regions where LiDAR data is present, producing a dataset concentrated in the global north and in places where LiDAR is commonly collected (landslide hotspots, flood zones, tectonically active regions), both of which would considerably bias our dataset.

9. Descriptions of many variables in the table 2 and 3 are incomplete and demand more details.

Thank you for highlighting this. We have added additional detail to variable descriptions in both tables, making direct reference to relevant equations, literature, and sections of the main text.

Reviewer 2

The manuscript introduces the GDBM, a near-global dataset of drainage basin morphology. It provides morphometric measurements of 254,966 drainage basins and their longest river channels, derived using SRTM topography data combined with Köppen-Geiger climate classifications. However, the current manuscript has several issues that must be addressed before it can be considered for publication.

We thank the reviewer for their consideration of our work and their suggestions on how to improve its clarity and impact. We believe that following these suggestions, the revised manuscript is markedly improved.

1. In March 2024, the journal ESSD published a paper titled "A global dataset of the shape of drainage systems." This work used global 90m DEMs to extract 0.67 million drainage basins larger than 50 km^2 and their longest river channels (from drainage divide to outlet). It also measured river length, sinuosity, basin area, and aspect ratio. The preprint of this article was available online in September 2023. I am puzzled and surprised as to why this highly relevant and important paper has been completely ignored. The authors should carefully compare the differences between the two studies and rewrite the second paragraph of the Introduction to better highlight the innovations of this paper based on He et al., 2024. I think this is this manuscript's most significant flaw.

This manuscript was drafted prior to *He et al.* [2024] being published, so we are grateful to you for drawing this relevant paper to our attention. We have added references to this paper into the introduction and have placed this new dataset into context alongside the others we already discuss so that readers can have a full appreciation of the merits of our dataset alongside the merits and use cases of other datasets that are available. The new second paragraph of the introduction reads:

> *With the increasing availability of high quality global and near-global topographic data, a number of compilations of global channel data have been produced, notably MERIT Hydro Yamazaki et al. [2019], HydroSHEDS Lehner et al. [2008], HDMA Verdin [2017], and Basin90m He et al. [2024]. These datasets are valuable resources for many avenues of research, typically however this existing family of datasets are not directly suited to analysis which links climate and channel morphometry. For some datasets, river mapping approaches make use of published blue line maps UK Centre for Ecology and Hydrology [2000], which perform well in humid environments but systematically exclude intermittent and ephemeral channels, particularly those found in drylands Jaeger et al. [2017]; Messager et al. [2021]. In the case of Basin90m He et al. [2024], rivers in drylands are removed based on their aridity, excluding many ephemeral channels from analysis. In other cases, morphometric data is extracted using HydroSHEDS as the input for basin extraction Shen et al. [2017], again potentially excluding intermittent and ephemeral channels from the dataset. Some*

datasets make use of a stream burning approach Yan et al. [2019] to enforce channelised flow across a DEM and this approach ensures a topologically consistent network, but modifies the topographic data, potentially biasing measurements of channel or basin morphology. Other datasets are predicated on lower resolution DEM products Lehner et al. [2008]; Lin et al. [2021]; He et al. [2024], reducing their ability to identify smaller channels. Several datasets do not record geomorphometric data natively Yan et al. [2019], requiring a user to load the channel data, source a DEM and then sample the required morphometric data. This lack of native morphometric information can lead to inconsistency and a reduction in the spatial scope of analyses. With the aim of addressing these limitations, supplementing existing datasets and facilitating the analysis of relationships between climate and basin morphometry, we present a new near-Global dataset of Drainage Basin Morphology, GDBM.

2. I suggest adding a section to discuss the limitations of the dataset, such as the exclusion of large basins and long rivers due to the division of DEMs.

We have added some further discussion of this point to the main manuscript, to ensure that readers are fully aware of how the dataset was generated, allowing them to make an informed decision about its appropriateness for their particular use case:

This division of the Earth's surface into a series of large tiles which can be processed in parallel is in line with other efforts to create large datasets of channel morphology He et al. [2024] and represents a compromise between computational power and dataset scale.

To ensure that each channel within GDBM corresponds to a distinct climate zone, drainage basins which cross or intersect with a climate sub-zone tile boundary are not recorded within the dataset (n=35,979). This filtering of data to exclude boundary crossing channels maximises the value of our dataset as a tool to explore relationships between climate and basin morphometry.

3. How do the authors verify that the rivers extracted from desert DEMs (e.g., in the Sahara Desert) are real and not artifacts such as dunes?

Due to the number of channels within the dataset, it would not be feasible to ground truth even a small subsample of them. However, our use of a conservative drainage threshold maximises the likelihood that the channels we identify are valid, as the probability of a true positive classification of a channel pixel increases as we move downstream. We also address these issues in our response to Reviewer One's fourth point, above. There have been extensive studies describing the interaction of fluvial systems and aeolian dunes [e.g., *Liu and Coulthard*, 2015] which suggests to us that the presence of channels within dryland systems is not in itself an indication of a problem with the dataset. One of the facets of this dataset is to ensure that we are capturing these ephemeral channels, which are often excluded from blue line maps, due to their intermittent flow. In an effort to understand if channels in drylands within our dataset were artefacts (i.e. have unexpected morphologies) we performed a sensitivity analysis as part of *Singer et al.* [2024], which is presented in Table S4 in that paper, and reproduced in the appendix of this response. This analysis excluded all channels from within dryland areas and found that the same morphometric patterns held, with or without these channels. This gives us further confidence that our data is reliable.

4. Please provide a global map of the rivers and basins included in this dataset.

Due to the number of channels within the dataset it is not practical to produce a global map of all of the rivers and basins within the dataset. At that scale the entire map is completely filled in and no detail can be identified. To illustrate this, we have produced a map where each channel in the dataset is represented by a single point, rather than its full length, and even with this simplification of the dataset, it is not possible to represent the data in a meaningful way:

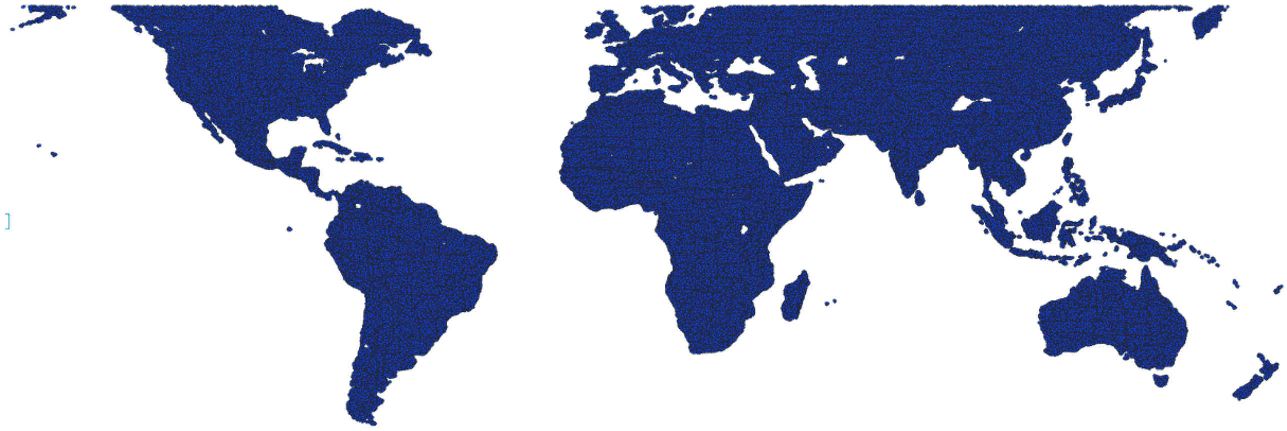

5. Providing only CSV files will limit the usability of this study for readers. Why are shapefile formats not included?

Due to the number of individual channels within the full dataset, it is impractical to provide the data in shapefile format, as each channel would be represented by 5 individual files, resulting in a considerable increase in computational overhead when attempting to analyse the dataset. We anticipate that the majority of users will interrogate these data programatically and so we have provided the data in the most appropriate format for that use case. The code within the repository, linked within the manuscript provides examples of how to convert data into shapefiles for visualisation.

6. Line 296: What does "CO2e" refer to?

CO_2e is the CO_2 equivalent of all greenhouse gases, and is used to represent all of the greenhouse gas emissions connected to a process, rather than solely CO_2 emissions. We have rewritten this as 'CO_2 equivalent' to make things clearer.

References

Chen, S.-A., K. Michaelides, S. W. D. Grieve, and M. B. Singer (2019), Aridity is expressed in river topography globally, *Nature*, *573*(7775), 573–577, doi:10.1038/s41586-019-1558-8.

Farr, T. G., P. A. Rosen, E. Caro, R. Crippen, R. Duren, S. Hensley, M. Kobrick, M. Paller, E. Rodriguez, L. Roth, D. Seal, S. Shaffer, J. Shimada, J. Umland, M. Werner, M. Oskin, D. Burbank, and D. Alsdorf (2007), The Shuttle Radar Topography Mission, *Reviews of Geophysics*, *45*(2), doi: https://doi.org/10.1029/2005RG000183.

He, C., C.-J. Yang, J. M. Turowski, R. F. Ott, J. Braun, H. Tang, S. Ghantous, X. Yuan, and G. Stucky de Quay (2024), A global dataset of the shape of drainage systems, *Earth System Science Data*, *16*(2), 1151–1166, doi:10.5194/essd-16-1151-2024.

Jaeger, K. L., N. A. Sutfin, S. Tooth, K. Michaelides, and M. Singer (2017), Geomorphology and sediment regimes of intermittent rivers and ephemeral streams, in *Intermittent Rivers and Ephemeral Streams*, pp. 21–49, Elsevier, doi:10.1016/b978-0-12-803835-2.00002-4.

Lehner, B., K. Verdin, and A. Jarvis (2008), New global hydrography derived from spaceborne elevation data, *Eos, Transactions American Geophysical Union*, *89*(10), 93, doi:10.1029/2008eo100001.

Lin, P., M. Pan, E. F. Wood, D. Yamazaki, and G. H. Allen (2021), A new vector-based global river network dataset accounting for variable drainage density, *Scientific Data*, *8*(1), doi:10.1038/s41597-021-00819-9.

Liu, B., and T. J. Coulthard (2015), Mapping the interactions between rivers and sand dunes: Implications for fluvial and aeolian geomorphology, *Geomorphology*, *231*, 246–257, doi:10.1016/j.geomorph.2014.12.011.

Messager, M. L., B. Lehner, C. Cockburn, N. Lamouroux, H. Pella, T. Snelder, K. Tockner, T. Trautmann, C. Watt, and T. Datry (2021), Global prevalence of non-perennial rivers and streams, *Nature*, *594*(7863), 391–397, doi:10.1038/s41586-021-03565-5.

Peel, M. C., B. L. Finlayson, and T. A. McMahon (2007), Updated world map of the Köppen-Geiger climate classification, *Hydrology and Earth System Sciences*, *11*(5), 1633–1644, doi:10.5194/hess-11-1633-2007.

Shen, X., E. N. Anagnostou, Y. Mei, and Y. Hong (2017), A global distributed basin morphometric dataset, *Scientific Data*, *4*(1), doi:10.1038/sdata.2016.124.

Singer, M. B., S. W. D. Grieve, S. Chen, and K. Michaelides (2024), Climatic controls on the length and shape of the world's drainage basins, *Geophysical Research Letters*, *51*(24), doi:10.1029/2024gl111220.

Trabucco, A., R. J. Zomer, et al. (2009), Global aridity index (global-aridity) and global potential evapo-transpiration (global-PET) geospatial database, doi:https://cgiarcsi.community/data/global-aridity-and-pet-database/.

UK Centre for Ecology and Hydrology (2000), UKCEH digital river network of Great Britain (1:50,000), doi:https://catalogue.ceh.ac.uk/documents/7d5e42b6-7729-46c8-99e9-f9e4efddde1d.

Verdin, K. L. (2017), Hydrologic derivatives for modeling and analysis—a new global high-resolution database, doi:10.3133/ds1053.

Yamazaki, D., D. Ikeshima, J. Sosa, P. D. Bates, G. H. Allen, and T. M. Pavelsky (2019), MERIT hydro: A high-resolution global hydrography map based on latest topography dataset, *Water Resources Research*, *55*(6), 5053–5073, doi:10.1029/2019wr024873.

Yan, D., K. Wang, T. Qin, B. Weng, H. Wang, W. Bi, X. Li, M. Li, Z. Lv, F. Liu, S. He, J. Ma, Z. Shen, J. Wang, H. Bai, Z. Man, C. Sun, M. Liu, X. Shi, L. Jing, R. Sun, S. Cao, C. Hao, L. Wang, M. Pei,

B. Dorjsuren, M. Gedefaw, A. Girma, and A. Abiyu (2019), A data set of global river networks and corresponding water resources zones divisions, *Scientific Data*, *6*(1), doi:10.1038/s41597-019-0243-y.

Zomer, R. J., A. Trabucco, D. A. Bossio, and L. V. Verchot (2008), Climate change mitigation: A spatial analysis of global land suitability for clean development mechanism afforestation and reforestation, *Agriculture, Ecosystems and Environment*, *126*(1-2), 67–80, doi:10.1016/j.agee.2008.01.014.

Appendix A

a) Results for all basins in GDBM

	Aridity Index category					K-G climate main zone				All
	Hyperarid	Arid	Semi-arid	Dry Subhumid	Humid	Tropical	Arid	Temperate	Cold	
n	19,196	47,897	51,220	24,065	112,573	61,366	97,016	42,267	54,302	254,951
**Median *L* (km)**	39.2	34.9	31.3	30.5	30.4	30.2	34.7	31.2	30.0	31.9
**Median *A* (km^2)**	235.2	219.6	213.6	218.9	218.3	215.6	222.5	218.8	216.7	218.9
**Median *GC***	2.739	2.532	2.336	2.250	2.333	2.340	2.515	2.262	2.331	2.384
**Hack's *h***	0.543	0.536	0.531	0.528	0.528	0.528	0.536	0.529	0.527	0.531

b) Results excluding basins in glacial regions at the LGM

	Aridity Index category					K-G climate main zone				All
	Hyperarid	Arid	Semi-arid	Dry Subhumid	Humid	Tropical	Arid	Temperate	Cold	
n	18,679	43,697	39,857	15,172	74,384	61,240	87,429	36,016	7,104	191,789
**Median *L* (km)**	39.3	35.3	32.2	31.5	30.6	30.3	35.3	31.8	30.7	32.7
**Median *A* (km^2)**	235.6	220.8	215.2	225.1	217.2	215.6	223.8	219.3	217.9	220.0
**Median *GC***	2.742	2.554	2.362	2.260	2.329	2.341	2.544	2.282	2.262	2.408
**Hack's *h***	0.543	0.537	0.532	0.529	0.528	0.528	0.537	0.530	0.528	0.533

c) Results for basins in glacial regions at the LGM

	Aridity Index category					K-G climate main zone				All
	Hyperarid	Arid	Semi-arid	Dry Subhumid	Humid	Tropical	Arid	Temperate	Cold	
n	517	4,200	11,363	8,893	38,189	126	9,587	6,251	47,198	63,162
**Median *L* (km)**	37.4	30.5	28.6	29.1	30.1	26.4	29.9	28.5	29.9	29.7
**Median *A* (km^2)**	228.3	206.3	207.5	210.2	220.5	228.3	210.9	216.7	216.5	215.8
**Median *GC***	2.641	2.270	2.243	2.232	2.342	1.939	2.249	2.144	2.341	2.305
**Hack's *h***	0.542	0.530	0.526	0.527	0.526	0.519	0.529	0.524	0.529	0.527

d) Results excluding basins in tectonic regions

	Aridity Index category					K-G climate main zone				All
	Hyperarid	Arid	Semi-arid	Dry Subhumid	Humid	Tropical	Arid	Temperate	Cold	
n	17,656	34,377	36,967	16,351	83,348	46,888	74,152	25,879	41,780	188,699
**Median *L* (km)**	39.4	35.8	32.2	31.0	30.4	30.1	35.7	31.7	30.4	32.4
**Median *A* (km^2)**	236.7	225.8	216.7	221.6	218.5	216.1	227.6	220.8	217.6	221.4
**Median *GC***	2.748	2.566	2.385	2.291	2.375	2.359	2.558	2.300	2.404	2.430
**Hack's *h***	0.543	0.537	0.532	0.529	0.527	0.527	0.537	0.530	0.528	0.532

e) Results for basins in tectonic regions

	Aridity Index category					K-G climate main zone				All
	Hyperarid	Arid	Semi-arid	Dry Subhumid	Humid	Tropical	Arid	Temperate	Cold	
n	1,540	13,520	14,253	7,714	29,225	14,478	22,864	16,388	12,522	66,252
**Median *L* (km)**	36.7	32.6	29.3	29.7	30.4	31.0	31.7	30.5	28.6	30.6
**Median *A* (km^2)**	216.9	203.6	205.4	214.0	218.1	213.6	206.6	215.4	213.8	211.7
**Median *GC***	2.625	2.425	2.202	2.162	2.199	2.275	2.356	2.188	2.105	2.241
**Hack's *h***	0.540	0.534	0.528	0.527	0.528	0.530	0.532	0.529	0.525	0.529

f) Results excluding basins in sand dune regions

	Aridity Index category					K-G climate main zone				All
	Hyperarid	Arid	Semi-arid	Dry Subhumid	Humid	Tropical	Arid	Temperate	Cold	
n	15,550	43,929	51,187	24,065	112,573	61,366	89,375	42,267	54,296	247,304
**Median *L* (km)**	37.2	34.4	31.3	30.5	30.4	30.2	34.0	31.2	30.0	31.6
**Median *A* (km^2)**	229.4	217.4	213.6	218.9	218.3	215.6	219.8	218.8	216.7	217.8
**Median *GC***	2.651	2.508	2.336	2.250	2.333	2.340	2.483	2.262	2.331	2.371
**Hack's *h***	0.538	0.535	0.531	0.528	0.528	0.528	0.534	0.529	0.527	0.530

g) Results for basins in sand dune regions

	Aridity Index category					K-G climate main zone				All
	Hyperarid	Arid	Semi-arid	Dry Subhumid	Humid	Tropical	Arid	Temperate	Cold	
n	3,646	3,968	33	0	0	0	7,641	0	6	7,647
**Median *L* (km)**	52.4	41.1	38.7	-	-	-	45.7	-	69.8	45.7
**Median *A* (km^2)**	260.8	245.5	253.4	-	-	-	252.6	-	206.8	252.6
**Median *GC***	3.393	2.846	2.558	-	-	-	3.072	-	3.860	3.073
**Hack's *h***	0.558	0.546	0.535	-	-	-	0.552	-	0.563	

h) Results for randomly sampled basins extracted with 25% lower area threshold (16.875 km^2)

	Aridity Index category					K-G climate main zone				All
	Hyperarid	Arid	Semi-arid	Dry Subhumid	Humid	Tropical	Arid	Temperate	Cold	
n	9,210	18,572	21,356	9,507	50,590	23,902	42,283	20,337	22,721	109,243
**Median *L* (km)**	33.7	30.8	27.3	26.8	26.2	26.3	30.2	27.0	25.8	27.7
**Median *A* (km^2)**	178.3	167.9	161.0	166.2	165.6	163.2	169.3	166.2	164.5	166.3
**Median *GC***	2.741	2.500	2.296	2.234	2.259	2.276	2.459	2.234	2.250	2.326
**Hack's *h***	0.544	0.537	0.531	0.529	0.527	0.528	0.536	0.530	0.526	0.531

i) Results for randomly sampled basins extracted with 25% higher area threshold (28.125 km^2)

	Aridity Index category					K-G climate main zone				All
	Hyperarid	Arid	Semi-arid	Dry Subhumid	Humid	Tropical	Arid	Temperate	Cold	
n	5,666	11,152	12,560	5,592	29,572	13,928	25,288	11,972	13,359	64,547
**Median *L* (km)**	44.8	39.3	35.2	34.6	34.1	34.3	39.1	35.2	33.3	35.9
**Median *A* (km^2)**	290.4	271.9	266.2	268.4	269.8	268.9	274.8	272.1	265.5	271.1
**Median *GC***	2.914	2.561	2.348	2.308	2.348	2.339	2.538	2.295	2.383	2.406
**Hack's *h***	0.546	0.537	0.531	0.530	0.528	0.528	0.537	0.530	0.527	0.532

j) Results for randomly sampled basins extracted with threshold of 1.69 km^2

	Aridity Index category					K-G climate main zone				All
	Hyperarid	Arid	Semi-arid	Dry Subhumid	Humid	Tropical	Arid	Temperate	Cold	
n	7,578	49,767	103,606	40,552	209,880	154,473	128,499	77,002	51,409	411,383
**Median *L* (km)**	9.2	9.0	8.3	7.9	7.9	8.0	8.6	7.8	7.8	8.12
**Median *A* (km^2)**	16.7	17.0	17.0	18.0	17.8	17.8	17.2	17.5	17.7	17.5
**Median *GC***	2.313	2.216	2.095	1.956	1.975	2.006	2.152	1.965	1.944	2.032
**Hack's *h***	0.54	0.537	0.531	0.526	0.526	0.527	0.534	0.526	0.526	0.529
